# No Apparent Workup for most new Indeterminate Pulmonary Nodules in US Commercially-Insured Patients

**DOI:** 10.36469/9674

**Published:** 2019-05-08

**Authors:** Bruce S. Pyenson, Carol M. Bazell, Michael J. Bellanich, Melissa A. Caplen, Javier J. Zulueta

**Affiliations:** 1 Milliman; 2 VisionGate 3D and Clinica Universidad de Navarra, Universidad de Navarra

**Keywords:** lung cancer screening, cancer treatment protocols, cancer screening tests, lung cancer, claims data analysis, indeterminate pulmonary nodules (ipn)

## Abstract

**Background:** A recent study estimated that more than 1.5 million Americans have an indeterminate pulmonary nodule (IPN) identified on a chest computed tomography (CT) scan each year outside of lung cancer screening programs. However, the cost and pattern of subsequent IPN workup have not been described for real-world settings.

**Objectives:** To examine the pattern and cost of IPN workup in real-world practice using insurer administrative claims data for commercially-insured individuals.

**Methods:** The primary source for this retrospective observational study was the MarketScan® 2013-2016 databases, which include information on 28 to 47 million insured lives. The newly diagnosed IPN study population consisted of members with an IPN diagnosis code on a claim in 2014 who did not have prior diagnosis of an IPN or lung cancer in 2013 and who had coverage from 2014 to 2016. Subsequent claims were examined for workups included in the American College of Chest Physicians (ACCP) guideline recommendations and the costs of workup were tabulated.

**Results:** Of the 15 064 patients in the study population, only 5471 (36%) received any subsequent workup. The average and median costs of workup for these patients were $3270 and $2068, respectively. Spread across the commercially-insured population, the workup is estimated to cost between $1 and $2 per member per year.

**Conclusions:** The majority of commercially-insured members with newly identified IPNs do not appear to have any guideline-recommended workup, despite a low incremental cost of such workup services on a population basis.

## Background

The widespread use of imaging to evaluate patient symptoms, coupled with required U.S. health insurance coverage of screening CT for patients at high risk for lung cancer, has increased the importance of the proper evaluation of radiologically-identified lung parenchymal abnormalities. Indeterminate pulmonary nodules (IPNs) are well-defined, non-calcified, nodules in the lung less than 3 cm in size^1^ that are completely surrounded by lung parenchyma. The primary goal in the evaluation of IPNs is to rule-out malignancy.

A recent study estimated that more than 1.5 million Americans have an IPN identified on a chest CT scan each year, not counting IPNs identified through lung cancer screening.^2^ This study examines the workup pattern of a large sample of these patients covered by commercial insurance. Of the 1.5 million identified IPNs each year, approximately 63 000 (4%) are estimated to receive a diagnosis of lung cancer within 2 years.

According to the American College of Chest Physicians (ACCP) guidelines, workup of an IPN is indicated unless there are specific nodule characteristics such as a benign pattern of calcification or the presence of fat within the nodule.^1^ The recommended workup depends on the probability of a nodule being a cancer before further workup (termed the pre-test probability), which can be determined by the physician’s judgment or through a validated model.^3^ For patients with a high pre-test probability of cancer (> 65%), the guidelines recommend a workup via surgical diagnosis unless specifically contraindicated. For patients with a low to moderate pre-test probability of cancer (5% to 65%), the guidelines recommend functional imaging (positron emission tomography (PET) or dynamic contrast enhancement CT). For patients with a very low pre-test probability of cancer (< 5%), active surveillance with a follow-up imaging test (generally, CT) is recommended. Active surveillance with periodic CTs at 3 to 6, 9 to 12, and 18 to 24 months is also recommended when the pre-test probability is low (<30% to 40%) and the results of a functional imaging test are negative, or when needle biopsy is nondiagnostic and the lesion is not hypermetabolic by PET.^1^ At least two studies of patients referred to pulmonologists and/or thoracic surgeons for evaluation of IPNs suggest that these specialists frequently do not follow the guidelines.^4,5^

Previous studies analyzed the cost^6^ and reductions in mortality^7^ associated with increased rates of lung cancer screening. Compared to screening for other cancers, lung cancer screening has a lower cost per life-year saved, due to the smaller at-risk population, the low cost of screening CTs, and high mortality associated with the late-stage diagnosis of lung cancer. The cost per life-year saved has been reported to be as low as under $20 000.^6^ However, there has been little analysis of workup of incidentally detected IPNs. The objective of this study was to analyze the pattern and cost of IPN workup in real-world practice using a very large database of insurers’ administrative claims.

## Methods

The study population of newly diagnosed IPN patients was derived from the MarketScan® database, which represents approximately 20 to 30 percent of the total employer-sponsored insurance market (as estimated by Kaiser^8^) and is widely used to represent the U.S., commercially-insured population.^9^ We identified the study population in 2014, using 2013 data to exclude patients with prior IPNs or cancer and 2015 and 2016 data to follow the study population. The study population consisted of patients who had a diagnosis code for solitary pulmonary nodule (International Classification of Diseases, Ninth Revision, Clinical Modification--ICD-9-CM--793.11) in any position on a qualifying claim during 2014; the first such claim in 2014 was the index claim. IPNs are well-defined, non-calcified, nodules in the lung less than 3 cm in size that are most commonly solitary; therefore, the specific diagnosis of a solitary pulmonary nodule on a claim likely represents an IPN rather than a lung abnormality of known etiology. We limited the study population to patients with no prior IPN or cancer diagnosis who were at least 18 at the time of IPN diagnosis and younger than 65 at the end of 2016 and who had continuous coverage through December, 2016.

We used the American Medical Association’s Current Procedural Terminology CPT® codes to determine whether an IPN patient had workups in the categories specified in the ACCP guidelines^1^: CT; PET; non- surgical biopsy and surgical resection. We calculated the allowed workup cost as the sum of amounts paid by the insurer plus any cost-sharing (such as a copay) paid by the patient.

Additional details on identifying the study population and workup costs are provided in the Technical Appendix.

For each category of workup, raw average costs and winsorized average costs (limiting the workup cost to no less than the 5^th^ percentile or more than the 95^th^ percentile of the workup’s category) were calculated. We constructed 95% confidence intervals around these averages assuming residuals were normally distributed. Total commercial population estimates were developed by applying the incidence rate to these estimates.

We used student’s t-tests for independent samples with unpooled variance to assess the differences in average age between the study and MarketScan populations. We used two proportion z tests to assess the differences in the percentages of females and former/current smokers between the study population and the MarketScan population.

We tested for differences in workup rates for patient age, sex, and smoking status using two proportion z tests. We calculated the average lag between initial diagnosis and workup and constructed 95% confidence intervals assuming residuals were normally distributed.

We performed a Kaplan-Meier analysis of workup rates on the newly diagnosed IPN population before removing patients without full enrollment to assess the impact of disenrollment (including death).

## Results

### IPN Study Population

From the MarketScan 2014 population of 23769995, we identified 52 828 members with an IPN diagnosis in 2014 and classified 36001 as new IPN patients (Table 1), for a 0.15% annual IPN incidence rate. Our IPN study population (“study population”) consisted of 15064 of the new IPN patients who had medical coverage in all months from the date of their IPN diagnosis through December 2016. The low portion of new IPN patients who had continuous coverage through 2016 is consistent with a 40% decline in MarketScan lives between 2014 and 2016 and normal insured population turnover.

Table 2 contains descriptive statistics for the MarketScan 2014 population and the study population. The study population was older than the MarketScan population (p<.01) and had a higher percentage of individuals with codes indicating that they were current or former smokers, (p<.01). The proportion of women in the study population (55.3%) was greater than in the MarketScan population overall (51.1%), (p<.01). The average per-person annual medical (non-pharmacy) spend for the study population was 440% greater than the MarketScan population ($19387 vs $3583), but actuarial age and sex factors^10^ accounted for only a 57% increase.

**Table 1.** Determination of Study Population

Population Requirements Number of Members	Requirements	Number of members	% of MarketScan Population
Total MarketScan 2014 Membership (Commerical market)	None	47258528	
	Active employee (or dependent) with 12 months of continuous enrollment in a non- 28350205 capitated plan in 2013	28350205	
MarketScan Population	Active employee (or dependent) enrollment in a non-capitated plan in January 2014	24625099	
	Age 64 or younger on 12/31/2016	23769995	100.00%
	IPN diagnosis (index event) in 2014	52 828	0.22%
	No IPN diagnosis in 12 months prior to 2014 diagnosis	45 402	0.19%
New IPN Patient Population	No cancer diagnosis in 12 months prior to IPN diagnosis	38 505	0.16%
	Active employee (or dependent) up to time of IPN diagnosis	37 854	0.16%
	Age 18 or older at the time of IPN diagnosis	37 466	0.16%
	No cancer diagnosis on date of IPN diagnosis	36 001	0.15%
**Study Population**	**Continuous coverage through December (end of study period)**	**15 064**	**0.06%**

**Table 2.** Descriptive Statistics for MarketScan and Study Populations

**Characteristics**	**MarketScan Population**	**New IPN Study Population**
Number of members	23 769 995	15 064
Age-band		
0-17	5 950 248 , 25.0%	- , 0.0%
18 - 34	5 917 333 , 24.9%	915 , 6.1%
35 - 44	4 079 950 , 17.2%	2362 , 15.7%
45 - 54	4 813 328 , 20.2%	5843 , 38.8%
55 - 64	3 009 136 , 12.7%	5944 , 39.5%
> 64	- , 0.0%	- , 0.0%
Average Age	33	50
Gender		
Male	11 634 010 , 48.9%	6728 , 44.7%
Female	12 135 985 , 51.1%	8336 , 55.3%
Region^1^		
Northeast	4 678 458 , 19.7%	3220 , 21.4%
North central	5 275 787 , 22.2%	3566 , 23.7%
South	8 426 106 , 35.4%	6205 , 41.2%
West	4 805 788 , 20.2%	2053 , 13.6%
Identified Current/Former Smokers	971 280 , 4.1%	3268 , 21.7%
Per member medical costs in 2014^2^	$3583.32	$19 386.90

^1^Census Bureau-designated United States region definitions ^2^Includes facility and professional allowed expenditures. Does not include pharmacy allowed expenditures.

### No Workup for the Majority of Incident IPN Patients

**Figure 1.**
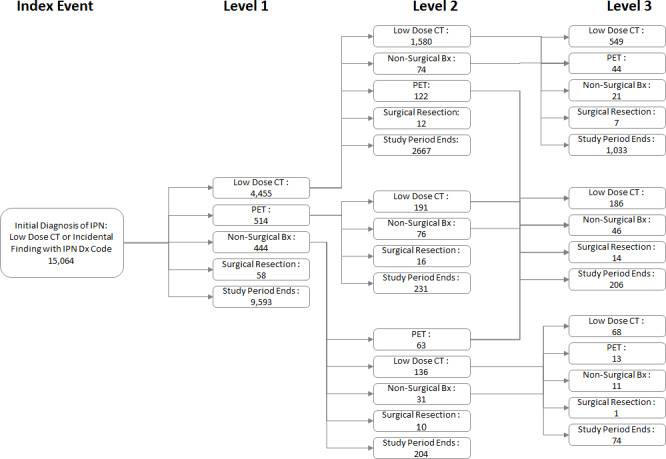
Path of IPN Population through Three Levels of Workup. Numbers Reflect Patient Counts

Figure 1 shows the workup paths and patient volumes for the study population. From the time of their IPN diagnosis in 2014 through the end of 2016, 36% of the IPN study population had an initial workup (Level 1), 15% secondary workup (Level 2), and 6% tertiary workup (Level 3). We found no Level 1 workup (or subsequent workup) for 64% of the study population. In Table 3, we show details for the Level 1 workup including smoking status, sex, and age. Patients’ dates of initial diagnosis were evenly distributed throughout 2014, and the average follow-up period was 30 months. Figure A1 in the Appendix shows that 80% of the study population had no workup within 180 days of their IPN diagnosis.

**Table 3.** Level 1 Workup

		**By Identified Smoking** **Status**		**By Gender**			**By Age**			
**Level 1 Workup**	**IPN** **Population (% of Total)**	**Current/ Former Smoker**	**Not Identified as Smoker**	**Male**	**Female**	**18-34**	**35-44**	**45-54**	**55-64**	
CT of thorax, without contrast	4455 (29.6%)	1107 (33.9%)	3348 (28.4%)	1880 (27.9%)	2575 (30.9%)	152 (16.6%)	584 (24.7%)	1693 (29.0%)	2026 (34.1%)	
PET	514 (3.4%)	166 (5.1%)	348 (3.0%)	224 (3.3%)	290 (3.5%)	17 (1.9%)	61 (2.6%)	176 (3.0%)	260 (4.4%)	
Non-surgical biopsy	444 (2.9%)	149 (4.6%)	295 (2.5%)	191 (2.8%)	253 (3.0%)	28 (3.1%)	61 (2.6%)	171 (2.9%)	184 (3.1%)	
Surgical resection	58 (0.4%)	20 (0.6%)	38 (0.3%)	25 (0.4%)	33 (0.4%)	3 (0.3%)	5 (0.2%)	21 (0.4%)	29 (0.5%)	
No workup^1^	9593 (63.7%)	1826 (55.9%)	7767 (65.8%)	4408 (65.5%)	5185 (62.2%)	715 (78.1%)	1651 (69.9%)	3782 (64.7%)	3445 (58.0%)	
**Total**	**15 064** **(100.0%)**	**3268** **(100.0%)**	**11 796** **(100.0%)**	**6728** **(100.0%)**	**8336** **(100.0%)**	**915** **(100.0%)**	**2362** **(100.0%)**	**5843** **(100.0%)**	**5944** **(100.0%)**	

^1^Members with no workup of any of the four types listed above within the study period.

While 44% of identified current or former smokers in the study population had any workup compared to 36% of the total study population (p<.01), over half (56%) did not receive any form of workup. Female patients were more likely to receive any workup (38%, p<.001), as were older patients (42% for patients aged 55 to 64, p<.001) compared to 36% of the total study population. For patients that had any workup, CT of the thorax without contrast was the most common workup of the four ACCP guideline categories. These CTs made up 81.4% of the Level 1 workup (initial workup) and 82.0% of all workups.

Patients with a workup had an average of 8 months between initial diagnosis and their first workup (Table 4). For patients with more than one workup, the average intervals between Level 1 and Level 2 workups and between Level 2 and Level 3 workups were about 8 months.

**Table 4.** Time Between Diagnosis and Workup

**Level 1 Workup**	**Number of Members**	**% of IPN Population**	**Average Months Between Dx and Level 1** **(95% Confidence Interval)²**	**Median Months** **Between Dx and Level 1²**
CT of thorax, without contrast	4455	29.6%	8.8 (8.5 , 9.0)	6.5
PET	514	3.4%	3.6 (3.0 , 4.1)	0.9
Non-surgical biopsy	444	2.9%	4.6 (3.8 , 5.3)	0.9
Surgical resection	58	0.4%	3.1 (1.8 , 4.3)	1.1
No workup¹	9593	63.7%	-	-
**Total**	**15 064**	**100.0%**	**7.9 (7.8 , 8.0)**	**5.7**

^1^Members with no workup of any of the four types listed above within the study period. ^2^Only reflects cases with distinct diagnosis and level 1 workup events. Cases where the initial IPN diagnosis was found on a non-surgical biopsy or surgical resection claim are excluded.

### Cost of IPN Workup

We calculated median workup costs of $549 for CT of the thorax, without contrast; $3328 for PET scan; $2794 for non-surgical biopsy; and $24623 for surgical resection. We calculated mean workup costs of $883 for CT of the thorax, without contrast; $3685 for PET scan; $7073 for non-surgical biopsy; and $32266 for surgical resection. The average total cost of IPN workup across the sequential workups (Levels 1, 2, and 3) experienced by a new IPN patient was $3270 as displayed in Table 5. The differential between median and mean workup costs resulted from a few high-cost outliers, some of which included services that were likely unrelated to the workup itself, such as chemotherapy following a surgical resection.

The average winsorized per IPN patient workup cost (all four categories) was $2702 95% CI ($2544-$2680). The non-winsorized average total workup cost was $3270 95% CI ($2954-$3587), and the median cost was $2068. Table 5 converts this IPN workup cost into the expected per-member-per-year cost for a typical commercial population. The winsorized estimate of the cost for the current level of workups of IPN for a commercial population was $1.49 per member per year (95% CI $1.40-$1.57).

**Table 5.** Cost of Workup

**Calculation of PMPM^3^ Workup Costs**	**Uncapped Cost Distribution^1^**	**Capped Cost Distribution^1,2^**	**Services Priced at Median**
Workup cost per patient with workup^1,2^	$3270 ($2954 , $3587)	$2702 ($2544 , $2860)	$2068
% of study population with workup	36.3%	36.3%	36.3%
Workup cost per patient	$1188 ($1073 , $1303)	$981 ($924 , $1039)	$751
New IPN incidence in commercial population	0.15%	0.15%	0.15%
Annual workup cost over total membership	$1.80 ($1.62 , $1.97)	$1.49 ($1.40 , $1.57)	$1.14
**PMPM^3^ Cost over total membership**	**$0.15 ($0.14 , $0.16)**	**$0.12 ( $0.12 , $0.13)**	**$0.09**

^1^ Quantities in parentheses represent the 95% confidence interval of the average workup cost ^2^ Each category of workup (CT, PET, non-surgical biopsy, and surgical resection) has been capped at 5^th^/95^th^ percentile to remove outlier services ^3^ PMPM – Per Member Per Month Cost

Approximately 37% of the winsorized total workup cost was derived from costs associated with CT of the thorax without contrast workups. Eighteen percent of the cost came from PET scans, 21% from non- surgical biopsy, and 24% from surgical resection. Approximately 69% of the workup cost was incurred within a year after the initial diagnosis, consistent with the finding that for those patients who received any IPN workup, slightly more than half (58%) had only one level of workup.

For all four categories of IPN workup (CT, PET, non-surgical biopsy, surgical biopsy), the workup itself, rather than the associated costs before or after the workup, made up 85% to 96% of the total IPN workup cost.

## Discussion

Two findings stand out from our analysis: the majority of individuals in the study group of patients with newly identified IPNs did not have any workup as specified in the ACCP guidelines, and the cost of IPN workups is low when considered on a population basis.

### Low IPN Workup Rates

The low workup rate of 36% we report is a concern because it may mean missed opportunities to detect lung cancer at an earlier and potentially curable stage, although we did not assess lung cancer incidence in this study. Even among those patients with a recognized history of smoking, the workup rate was only 44%. We report an incidence rate for newly diagnosed IPNs of 0.15% in the 18 to 64 active employee/dependent MarketScan population, which is half the approximately 0.28% incidence rate estimated from Gould^2^ for the 18-64 population. Our lower incidence is not unexpected, as the Gould study used information from electronic medical records, which likely contained highly detailed findings not deemed relevant to the payment of claims. Claims-based studies such as ours often under-identify diagnoses, and such under- identification is often associated with a bias to identifying more severe cases.^11^ This makes our low workup rate even more concerning.

It is possible that further IPN workup may not have been clinically indicated for some new IPN patients in our sample. We examined whether patients without further IPN workup had IPN-related office visits after initial IPN diagnosis, because at that office visit a provider may have recommended that the patient obtain no follow up. We found that only 15% of the study population without any IPN workup had an office visit with an IPN diagnosis code following the initial IPN diagnosis. In these cases, the provider may have recommended no additional workup. Even if all of such office visits were accompanied by a recommendation of no further workup, the non-workup rate would fall only to 54% (64% x (1-15%)) -- still a surprisingly high proportion.

ACCP guidelines recommend that even low-risk IPN patients should receive active surveillance that includes follow-up CT.^1^ Therefore, we would expect most new IPN patients to have a chest CT or more intensive category of IPN workup following the initial IPN diagnosis. The low workup rate seen in our analysis is consistent with other study results that have shown that guidelines for the workup of IPNs are not consistently followed. The Fleischner Society has published guidelines for the workup of pulmonary nodules,^12^ but adherence by radiologists is as low as 34%.^13^ Evidence shows that when guidelines are not followed, over- and under-evaluations of lung nodules are common.^14^ Over-evaluation can occur in up to 20% of cases and leads to prolonged surveillance with increased radiation exposure, invasive biopsies, anxiety, and unnecessary surgeries. In a multicenter study on the clinical utility of a bronchial genomic signature for the diagnosis of lung cancer in individuals referred for a bronchoscopy for a suspicious nodule or lung mass, 50% of patients who underwent a surgical biopsy had a final diagnosis of benign disease, which indicates over-evaluation and over-management.^15^ Furthermore, 20% of the patients diagnosed with lung cancer in the study received the diagnosis between 3 and 12 months after the bronchoscopy (under-evaluation). Under-evaluation occurs in up to 27% of patients, resulting in the possibility of delayed diagnosis and a missed chance for curative surgical treatment.^1^

In a study of 377 patients with pulmonary nodules (8-20 mm in diameter) evaluated by community pulmonologists, 10% were at low risk, 80% were at moderate risk, and 10% were at high risk for malignancy.^5^ The rate of surgical resection was similar among the three risk groups (17%, 21%, and 17% respectively; p=.69), and 35% of the surgeries had a final diagnosis of benign disease. In another study of 337 subjects, 52% of patients with an IPN and a very low pre-test probability of cancer (<5%) were managed more aggressively than the guidelines recommend, mostly with a PET scan instead of serial CTs at intervals of 6-12 months as recommended by ACCP for this population.^4^ In the latter study, the conditional prevalence of cancer in each group of IPN patients with an estimated pre-test probability of malignancy was 12% among very low risk (<5%), 23.6% among low to moderate (5-65%), and 82.6% among high risk.^4^ Thus, in the low risk group the prevalence of cancer was greater than the estimated pre-test probability.

While the low IPN workup rate we found is consistent with the studies showing under-evaluation of nodules and lack of adherence to guidelines, the rate of non-surgical biopsies and surgical resection as a proportion of workup we observed is lower than the results of other studies. We found that 14% of the new IPN patients that received any workup had a non-surgical biopsy or surgical resection. For comparison, Tanner found that 17-20% of patients, regardless of lung cancer risk category, had a surgical resection as their most invasive test.^5^ However, Tanner studied patients referred to pulmonologists’ practices for evaluation of IPNs, while our study included the full population of patients with newly diagnosed IPNs.

The patients in Tanner’s study may have had prior workup or otherwise been perceived as at elevated risk for lung cancer.

Our claims-based methodology did not allow us to assess patient lung cancer risk, which would include factors such as nodule size and pack-years of smoking, so we are unable to compare the distribution of risk categories in the two study populations.

### Cost of Workup

We found that current IPN workup spread across the entire commercially insured population cost $1.49 (95% CI $1.40-$1.57) per member per year, assuming no cost-sharing. This was very low cost as a percent (less than 0.1%) of nationwide spending on healthcare for commercial populations; in 2014 the average cost for an employer that provided health benefits to a single employee was $4598.^16^ If we assume that the 64% of IPN patients who did not receive any IPN workup in our sample received the average pattern of IPN workup we observed for patients who received any workup, the additional $3 per member per year would still be under 0.1% of the average cost of commercial payers’ claims.

These results show that providing workup for all IPNs we identified would be lower cost, on a population basis, than the cost associated with lung cancer screening shown in other studies.^6^ However, it is not known what proportion of new IPNs identified were found among people who should have been screened for lung cancer.

We note that our study population of newly-diagnosed IPN patients had much higher overall annual claim costs, an approximately 245% differential after accounting for age-sex mix. This suggests the study population had much higher morbidity than the MarketScan population, which is consistent with respiratory or other symptoms and the resulting diagnostic tests that could lead to an IPN diagnosis.

## Limitations

Our retrospective observational study used administrative data, and this methodology has advantages and disadvantages compared to studies that use medical records or randomized controlled trials. Claims data allow the examination of the full scope of services that may be performed in all sites of care for large numbers of patients. Our source database contained full claims information on tens of millions of lives from 2014-2016, so is likely more representative of recent national practice patterns than either clinical trials or studies based on electronic medical records. Almost by definition, payer costs are best captured through administrative data, because administrative data directly generates the spending that appears in payers’ audited financial statements. However, claims data lack clinical information such as nodule size, nodule radiographic characteristics, and smoking history on all patients, which would be useful in determining the IPN patient’s probability of cancer and assessing whether treating physicians follow clinical guidelines for IPN workup. Future research is needed to identify lung cancer incidence among patients with no workup—and the harms of delayed diagnosis. Furthermore, the diagnoses and procedures that appear in administrative data may be incomplete and may include errors.^17,18^

Identifying new IPN patients using a claims-based algorithm that relies on diagnosis codes is the only feasible approach to analysis of medical conditions, patterns of care, and cost in large populations or where electronic medical records or other comprehensive sources of data are not available. Such claims-based techniques are strongly favored for population-level quality metrics and are integral to programs that affect billions of dollars of insurers’ and providers’ payments, such as HEDIS and STAR ratings.^19,20^ We note that the demographic characteristics of new IPN patients were similar to the characteristics of patients with newly identified IPNs in a large integrated health system.^2^ Specifically, we found that new IPN patients were more likely to be female, older, and current or former smokers, consistent with findings from that study. This consistency increases our confidence that individuals we identified as new IPN patients were likely to need workup.

For simplicity, we required patients to have complete enrollment to the end of the study period so that we could observe all workups during the study duration. However, this condition means we excluded patients who left the MarketScan database, including those who may have died of lung cancer during the average 2.5 years of follow up after the initial IPN diagnosis. We tested the impact of this full-exposure requirement on non-workup rates by producing a Kaplan-Meier estimate of non-workup rate for the newly diagnosed IPN population before the full-exposure requirement. This produced a non-workup rate of 61% compared to 64% for the study population, which suggests that our simplified approach to the follow up period did not have an important impact on the study results. Additional details on the results of the Kaplan-Meier analysis are provided in the Technical Appendix.

The extent to which patients diagnosed with IPNs but without further workup are later diagnosed with lung cancer is important but was not included in our analysis, but estimates would be possible with further research on claims databases. The diagnosis of an IPN that is not followed by a workup may represent a missed opportunity for the earlier diagnosis of lung cancer. It has been shown that the low rate of lung cancer screening among high-risk individuals contributes to the high proportion of late-stage lung cancers at cancer diagnosis and consequent dismal lung cancer survival rate.^21^ Identification of IPNs is a necessary, but not sufficient, step in the earlier diagnosis of lung cancer. Without workup of the IPN, its identification alone will not result in earlier lung cancer detection.

The low rate of workup for newly diagnosed IPNs is potentially alarming, and future research is needed to quantify the cost, mortality, loss of quality of life, and health impact of this non-workup. As lung cancer screening becomes more widespread, persistently low real-world IPN workup rates will interfere with early- stage lung cancer detection, the major benefit of screening. Research into patient- and clinician-focused strategies to improve real-world IPN workup rates through more consistent adherence to IPN workup guidelines, as well as future innovations in laboratory-based tests for IPNs, may contribute to improving the low real-world IPN workup rates.

## Author Contributions

BSP made key contributions to the study design, assisted with interpretation of analysis results, and helped draft the manuscript. CMB made key contributions to the study design, assisted with interpretation of analysis results, and helped draft the manuscript. MJB participated in the study design, assisted with interpretation of analysis results, assisted with performing the technical analysis, and helped draft the manuscript. MAC performed the technical analysis, assisted with interpretation of analysis results, and helped draft the manuscript. JJZ made key contributions to the study design, assisted with interpretation of analysis results, revised the submitted article for important intellectual content. All authors read and approved the final manuscript. They are accountable for all aspects of the work and in ensuring that questions related to the accuracy or integrity of any part of the work are appropriately investigated and resolved.

## Competing Interests

Bruce Pyenson, Carol Bazell, Michael Bellanich, and Melissa Caplen are employees of Milliman, Inc. in New York. Javier Zulueta is an employee of VisionGate 3D.

## Funding

Milliman, Inc. was commissioned by VisionGate 3D to produce this study.

## Acknowledgments

We would like to acknowledge Christine Ferro, Jared Hirsch, Noah Champagne, and Kate Fitch of Milliman, Inc. for their technical contributions.

## Figures and Tables

**Figure:** Supplementary Content
